# LegioTyper: Rapid typing of *Legionella pneumophila* serogroup 1 by flow-based chemiluminescence sandwich microarray immunoassay

**DOI:** 10.1007/s00216-025-06194-3

**Published:** 2025-11-05

**Authors:** C. Bärwinkel, A. Petzold, C. Lück, M. Petzold, M. Seidel

**Affiliations:** 1 Chair of Analytical Chemistry and Water Chemistry, School of Natural Sciences, Technical University of Munich, Lichtenbergstraße 4, 85748 Garching, Bavaria Germany; 2 Institute of Medical Microbiology and Virology, University Hospital Carl Gustav Carus, Medical Faculty, Dresden University of Technology, Fiedlerstr. 42, 01307 Dresden, Germany

**Keywords:** CL-SMIA, Serotyping, ELISA, Microarray, Chemiluminescence, ELISA, Serotyping, Legionella

## Abstract

Chemiluminescence imaging of a sandwich microarray immunoassay (CL-SMIA) is a simple and rapid method for antibody-based serotyping of pathogens like *L. pneumophila*, which is important to identify even outbreak-associated subtypes like *L. pneumophila* Sg 1 subtype Philadelphia. The CL image pattern can be interpreted by a decision path similar to the well-known “Dresden Panel”, which is typically performed via indirect ELISA. New antibodies needed to be characterized for the so-called LegioTyper panel as capturing antibodies to perform the flow-based CL-SMIA. The following was achieved for the LegioTyper principle: (i) minimizing the costs for antibody microarrays by manufacturing Jeffamine-2003-coated polycarbonate sheets, (ii) optimized automated test procedure which is able to perform the CL-SMIA in 34 min, and (iii) covalent immobilization of cAb using a microdispensing buffer which contains reactions for stabilizing and bioconjugation of cAb. Nine mAb and one polyclonal Ab (pAb) were characterized regarding the selectivity to *L. pneumophila* Sg 1 and additionally to the virulence-associated subtypes Knoxville, France, Allentown, Benidorm, and Philadelphia (all Pontiac group), OLDA, Oxford, Bellingham, Heysham, and Camperdown (all non-Pontiac group). We could show that by using the LegioTyper it is possible to differentiate between *L. pneumophila* Sg1 and non-Sg1 and between Pontiac and non-Pontiac strains. Additionally, subtyping with the “LegioTyper Panel” was possible for most of the outbreak-associated subtypes. Overall, our study showed that the LegioTyper principle can be used for rapid antibody-based subtyping of *L. pneumophila* Sg 1 after cultivation. It is an alternative to indirect ELISA or qPCR, which are more labor- and cost-intensive, and the automation is more complicated.

**Graphical Abstract:**

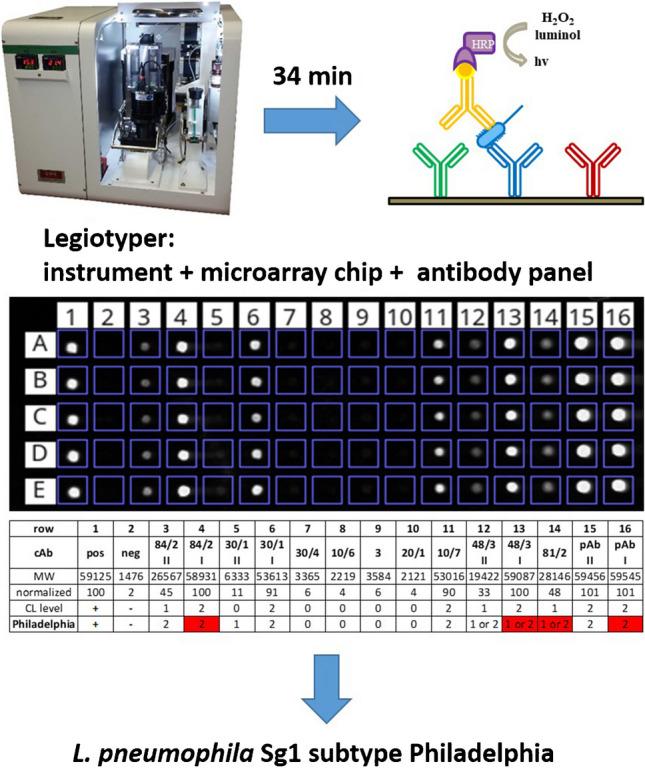

**Supplementary Information:**

The online version contains supplementary material available at 10.1007/s00216-025-06194-3.

## Introduction

Research on rapid testing methods for pathogens is of substantial importance, addressing the one health concept bridging the link between environmental and human or animal health. The Legionellosis outbreak is one of the most important examples where innovative bioanalytical methods are needed for improved outbreak management. As *Legionella* spp. can be found in various sources, it became necessary to compare clinical strains (often isolated from the lower respiratory tract, like bronchoalveolar lavage, sputum) with environmental strains by bioanalytical methods (molecular biological or antibody-based) to identify the source of infection.

*Legionella* spp. are rod-shaped, gram-negative bacteria with a monotrich flagellation and a length of approximately 2 to 5 µm. They inhibit natural and artificially created freshwater systems, soils, and biofilms. *Legionella* occur often as intracellular parasites of autotrophic protozoa, for example, in the genera *Acanthamoeba* and *Naegleria* [1–3], which fulfill their nutrient requirements such as *L*-cysteine and iron salts [4, 5]. Biofilms, frequently found on materials like plastics in sanitary engineering [1], promote *Legionella* proliferation under conditions of stagnation, lime deposits, or insufficient decontamination [1]. More than 60 different *Legionella* species and more than 70 Sg are known till present, classified as potentially human pathogenic [6] and cause the so-called legionellosis, an atypical pneumonia, with a lethality rate of up to 10% [7]. The greatest risk of infection is posed by the species *Legionella pneumophila*, which causes 90% of legionellosis infections [7]. In more than 80% of the cases, Sg 1 of this species occurs [8]. Infection occurs via inhalation or aspiration of contaminated bioaerosols that are evaporated from technical water systems like cooling systems, cooling towers, spas, or showers, leading to intracellular replication in macrophages [2].

In the case of an outbreak, more than one patient suffers from Legionnaires’ disease. Recent outbreaks worldwide have been attributed to aerosol-emitting systems such as evaporative cooling systems [9, 10]. However, the majority of cases often mislinks to common sources, urging a more unbiased approach during source attribution investigations [11, 12]. During outbreaks, rapid identification of the infection source, like evaporative cooling systems, cooling towers, wet separators, car washes, whirlpools, dental units, etc., is crucial. The diversity of environmental sample matrices complicates direct comparison to clinical samples. In an outbreak management plan, it is defined which different water samples have to be analyzed [13]. The huge number of water samples is one reason why rapid serotyping of *Legionella pneumophila* is recommended as an alternative method to cultivation for source identification [14–16].

Serotyping of bacteria commonly involves immunological detection methods using antibodies, which are fast and cost-effective because no additional equipment is required. *Legionella* antigens are lipopolysaccharide (LPS) structures located on the outer membrane [17–19]. These structures form the basis of the phenotypic analysis of *L. pneumophila*, differentiating this species into 15 Sgs and additional subgroups of Sg 1 using monoclonal antibodies targeting the LPS. The so-called Dresden Panel is therefore an important tool of epidemiological typing methods for the comparison of clinical and environmental isolates. Antibody-based serotyping of legionellae is performed by an indirect immunoassay such as ELISA or immunofluorescence [20].

In this study, we established the LegioTyper analysis platform as a rapid and automated test for the identification and subtyping of *L. pneumophila* Sg 1 from culture samples. The LegioTyper consists of (i) a panel of capturing antibodies (cAb), (ii) a simple production strategy of Jeffamine-coated polycarbonate microarray chips, (iii) a flow-based CL-SMIA automatically processed by the microarray chip reader (MCR-R), and (iv) a ternary code to interpret digitally the CL image pattern of each subtype of *L. pneumophila* Sg 1. A total of nine monoclonal cmAb and one polyclonal cpAb were used for the multiplexed subtyping of *L. pneumophila* Sg 1. This new “LegioTyper Panel”, which is based on the well-established “Dresden Panel”, was evaluated with heat-inactivated subtypes of *L. pneumophila* Sg 1 at concentrations of 10^7^ cfu mL^−1^. The automated CL-SMIA takes 34 min and is much faster and simpler to perform than the usually used indirect ELISA methods. We could show that with the LegioTyper it is possible to differentiate between *L. pneumophila* Sg 1 and non-Sg 1 and between the more virulent Pontiac and non-Pontiac strains. The subtyping with the “LegioTyper Panel” was possible for most of the subtypes Philadelphia, Benidorm, Heysham, Bellingham, and Camperdown. The LegioTyper system is a simple and fast method for serotyping of *L. pneumophila* and could be further improved by adding cmAb to differentiate also between the subtypes and even other serogroups.

## Materials and instrumentation

### Chemicals and materials

All chemicals were purchased from Sigma-Aldrich (Taufkirchen, Germany) unless stated otherwise. Jeffamine® ED-2003 was obtained from Huntsman Corporation (Osnabrück, Germany). Sodium hypochlorite for the decontamination solution was purchased from Carl Roth (Karlsruhe, Germany). Poly-HRP-Streptavidin was purchased from Senova (Jena, Germany) and the CL reagents luminol and hydrogen peroxide from Cyanagen (Bologna, Italy). The purified and biotin-labeled polyclonal antibodies were obtained from Meridian Life Science (Memphis, TN, USA) via the distributor Dunn Labortechnik GmbH (Asbach, Germany). Furthermore, the mAb was purified by Sifin diagnostics GmbH (Berlin, Germany). For all experiments, ultrapure water was used, unless mentioned otherwise. The phosphate-buffered saline (PBS) buffer consisted of 145 mM NaCl, 70 mM K_2_HPO_4_, and 10 mM KH_2_PO_4_. Running buffer contained 0.5% (w/v) casein and 0.01% Pluronic F127 dissolved in PBS buffer.

### Bacterial strains and culture conditions

To represent the broad spectrum of water bacteria that inhabit similar or identical niches like *Legionella* spp., we extended the list of bacteria that needed to be tested for the validation of qPCR test systems to identify *L. pneumophila* in water samples (ISO/TS 12869:2019 [21]) (see suppl. material Table 1 and suppl. material Table 2). We also included bacteria that are frequently found in urine samples as well as commercial skin bacteria. Bacterial strains (other than legionellae) are obtained from cryo stocks, plated on Colombia blood agar from Xebios Diagnostics GmbH (Düsseldorf, Germany), and incubated for 16–28 h at 37 °C. *Legionella* spp. isolates were plated on GVPC agar from Xebios Diagnostics GmbH (Düsseldorf, Germany) and incubated for 2 to 7 days at 37 °C. Bacteria colonies are harvested in autoclaved, ultrapure water. Fractions of the bacteria suspension were either stained with SYBR Green 1 from Biozym Diagnostik (Oldendorf, Germany) and quantified by flow cytometry (FCM) using BD Accuri™ C6 plus from BD (Heidelberg, Germany) or stained with Syto 9 from Sysmex (Bornbach, Germany) and quantified by FCM using the CyFlow® Cube6 also from Sysmex.

### Indirect ELISA

#### Antibodies

The antibodies against *L. pneumophila* were produced as described elsewhere [19]. Supernatants of hybridoma cell lines were purified by Sifin Diagnostics GmbH (Berlin, Germany) by affinity chromatography and conserved with 0.9% NaN_3_ per 10 mg antibody. Antibody isotypes were specified using the mouse isotyping kit (BioRad, Hercules US-CA). As detection and capture antibodies, three polyclonal antibodies were tested: PA1-7227 (Thermo Fisher, Rockford, US-IL); B65051B (Meridian Life Science, Memphis, US-TN); and OBT0943 (BIO-RAD, Feldkirchen, Germany) for their reactivity with *Legionella* spp. antigen.

#### Enzyme-linked immunosorbent assay (ELISA) for antibody evaluation

For each bacterium, three cell suspensions of 10^8^ cells mL^−1^ in PBS were prepared. Each suspension was tested native and heat inactivated (95 °C for 15 min). Cavities of a Greiner medium binding clear 96-well plate with a flat bottom from Sigma Aldrich (Munich, Germany) were coated with 50 µL of the cell suspension. The plate was incubated overnight in a humid and dark chamber at 4–8 °C or at 37 °C for 2 h. After washing the cavities three times with PBS, 200 µL blocking buffer (10% fetal calf serum (FCS) in PBS) was added into each cavity and incubated in darkness for 30 min at 37 °C. Subsequently, the cavities were washed three times with PBS. 50 µL of the primary mAb was added and incubated as described before. After a further washing cycle with PBS, 50 µL of 1:800 *anti*-mouse conjugated horseradish peroxidase (HRP) from Merck KGaA (Darmstadt, Germany) diluted in PBS was added, followed by incubation for 90 min at 37 °C in darkness. After a washing cycle, 50 µL of 3,3′,5,5′-Tetramethylbenzidine from Seramun Diagnostica GmbH (Heidesee, Germany) was added. The reaction was stopped with 1 M HCl after 10 min incubation in darkness. The cavities were read out at 450 nm and 650 nm using the Power Wave 200 microplate spectrophotometer (BioTek, Winooski, US-VT). The negative control cavity should have an OD_delta 450/620_ < 0.1. Results with OD_delta 450/620_ > 0.5 are positive [+], > 1 clearly positive [+++]. Values between 0.5 and 1 are intermediate [++]. Only clear positive results were regarded as valid for a subgroup specific reactivity.

### CL-SMIA

#### Preparation of antibody microarray chips

Polycarbonate microarray chips were prepared as described elsewhere [22]. One clear 1.0 mm thick polycarbonate slide from Modulor (Berlin, Germany) was cut into a 3 × 3 chip format with a single chip size of 76 mm x 26 mm using the digital cutting plotter CE600-40 from Graphtec Corporation (Yokohama, Japan). The slides were coated with succinic anhydride modified Jeffamine ED-2003 with a screen printer HDT150 from Siebdruckversand (Magdeburg, Germany) and incubated for 2 h at 100 °C. Afterwards, the slides were removed and cleaned twice for 15 min with ultrapure water in an ultrasonic bath, dried under a nitrogen flow, and stored in a drying box until usage. Antibody microarrays were produced by microdispensing cAbs in a grid of five rows of each cAb by contact printing using the BioOdyssey Calligrapher MiniArrayer from Bio-Rad Laboratories (Munich, Germany) and the corresponding solid pin SNS9 from Arraylt (Sunnyvale, USA). The contact printer was loaded with the coated 3 × 3 chip format and a 365-well plate containing the antibodies depending on the test series. The antibodies were 1:1 mixed with the spotting buffer to reach a final concentration of 1.0 mg mL^−1^*N*-(3-Dimethylaminopropyl)-*N*′-ethyl-carbodiimide hydrochloride (EDC), 1.0 mg mL^−1^*N*-hydroxysulfosuccinimide sodium salt (S-NHS), 0.01 mg mL^−1^ Pluronic F127, and 50 mg mL^−1^ Trehalose in 1 × PBS. As positive control, anti-HPR antibody produced by Sigma-Aldrich and, as negative control, spotting buffer were used. The spotting procedure was performed at 25 °C and 55% humidity, and the 3 × 3 chip format was afterwards incubated overnight at the same conditions. The chips were divided into the single parts and assembled using double-faced adhesive foil containing the flow channel and black polyoxymethylene carriers. The microarrays were afterwards filled with 1% bovine serum albumin in 1 × PBS solution for blocking, sealed with Rotilabo® sealing-films from Carl Roth (Karlsruhe, Germany), and stored at 4 °C until usage.

#### CL-SMIA on analysis platform MCR-R

The CL-SMIA assay was performed on the fully automated microarray chip reading platform MCR-R [23, 24] manufactured by GWK Präzisionstechnik GmbH (Munich, Germany). Prior to use, the system was flushed twice using running buffer containing 0.5% casein and 0.01% Pluronic F127 in 1 × PBS. Afterwards, two plastic syringes were filled with 0.02 µg mL^−1^ biotin-labeled detection antibody and 2.0 µg mL^−1^ poly-HRP-streptavidin solution using running buffer, and the syringes were attached to the device, respectively. The beakers for the CL reagents luminol and hydrogen peroxide were also filled, and the loading program was started to process the CL-SMIA by MCR-R. Prior to every measurement, the microarray chip was inserted into the system, flushed with 5000 µL running buffer at 500 µL s^−1^ to remove the blocking solution, and a dark frame image was made. Afterwards, the measurement could be started by injecting 600 µL sample into the sample loop using a 1.0 mL syringe, and the MCR-R fully automatically performed the CL-SMIA measurement. The sample was transported to the chip with 140 µL running buffer at 50 µL s^−1^ and afterwards traversed the chip in stop-flow mode with 1.0 µL s^−1^. Therefore, after every 60 µL, an incubation time with no flow of 30 s was conducted, and these steps were repeated until the whole sample passed the chip. After every single incubation step, the chip was washed with 1500 µL running buffer, respectively. In the same stop-flow mode, 600 µL biotinylated detection antibody were used with 2.0 µL s^−1^ and 5 s incubation time, and afterwards 600 µL poly-HRP labeled streptavidin were transferred over the chip. The CL reaction was started by mixing 200 µL luminol with 200 µL hydrogen peroxide and flushing afterwards over the chip with 100 µL s^−1^. During the CL reaction, an image was taken by a CCD camera for 60 s, which was displayed on the screen as the result of the CL-SMIA measurement. Before starting a new measurement, the sample loop was rinsed with 10 mL running buffer. At the end of a measurement day, the MCR-R was decontaminated for shutdown. Therefore, all syringes and the CL reagent beakers were filled with ultrapure water, and 0.1% sodium hypochlorite, 0.2% sodium thiosulfate solution and ultrapure water were connected successively for decontamination and cleaning. For data evaluation, the detected signals were corrected using the dark frame image and were stored as txt files. Using the evaluation software MCR spot reader (Stefan Weißenberger, Munich, Germany), a grid was placed, defining the position of the spots. For each spot, the ten brightest pixels were used to calculate the CL signal, and the mean value and standard deviation were calculated for five replicates per row. Spots deviating more than 10% from the mean value were excluded to a maximum of two spots per row.

### LPS antigen and urine sample preparation

For LPS spiking experiments, the LPS was isolated and purified as described elsewhere [24]. *Legionella* were freshly cultivated and cell concentration determined was adjusted to 10^10^ cells mL^−1^ as described above. For LPS shedding, the cell suspension was shaken for 14 h at 850 rpm and 37 °C, and afterwards centrifuged for 10 min at 4000 × g, and the supernatant filtered (0.2 μm cellulose acetate filter) to obtain the soluble LPS. In addition, human urine specimens (stored at −70 °C) were used as well. These samples were submitted to the laboratory as part of routine practice and were intended for the detection of *Legionella pneumophila* antigen at the National Consultant Laboratory in Dresden. The data were anonymized prior to analysis. In order to concentrate the urine samples’ volume, ultrafiltration was used. Therefore, the urine samples were concentrated with a Vivaspin centrifugal concentrator MWCO 5 kDa from Sigma-Aldrich (Taufkirchen, Germany), and afterwards the samples were injected directly into the LegioTyper instrument.

## Results and discussion

### Flow-based CL-SMIA principle

**Fig. 1:**
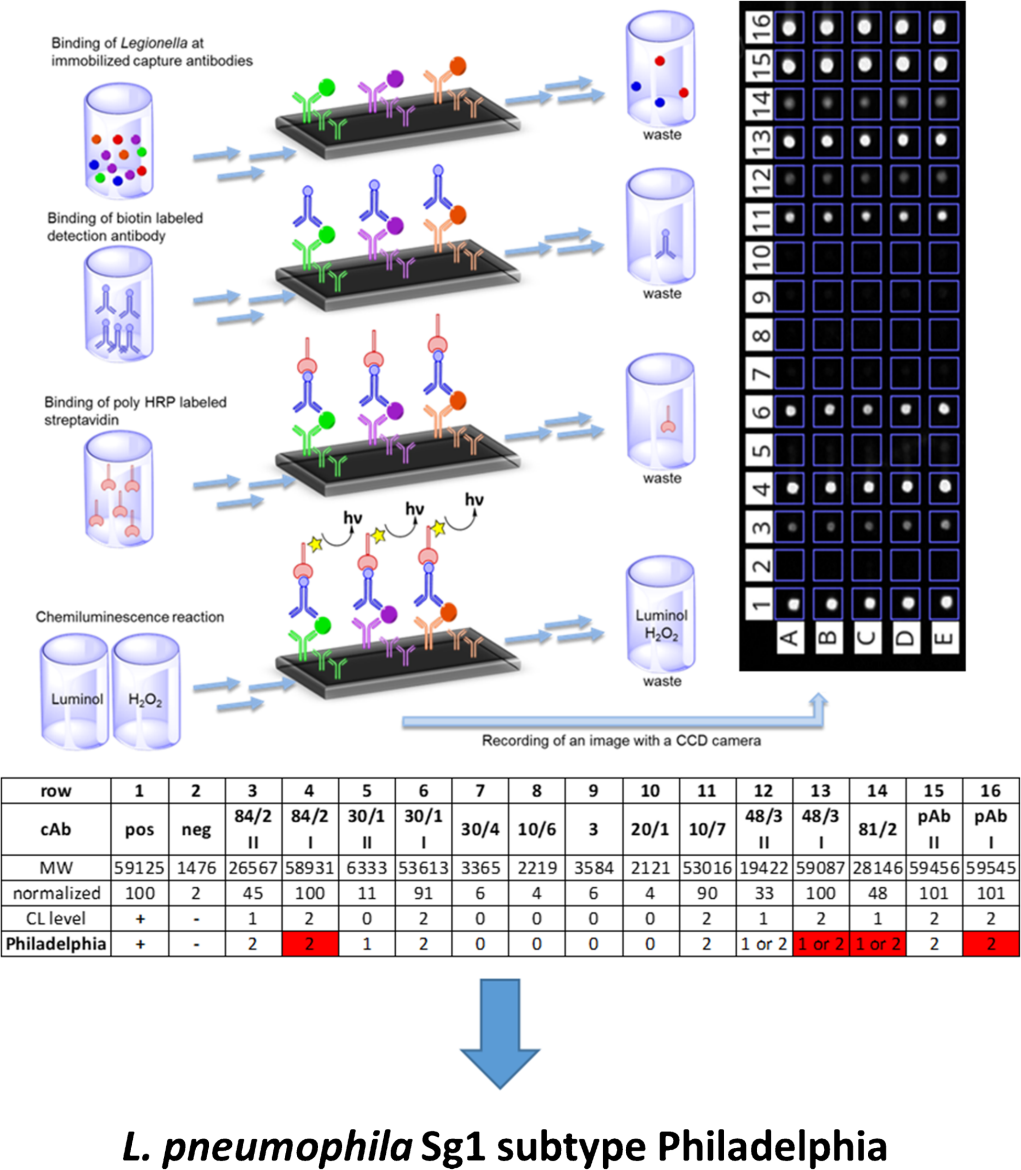
Principle of CL-SMIA creating a CL image pattern for each cAb on polycarbonate microarray chips, which is interpreted by a ternary code to identify subtypes of *L. pneumophila* Sg 1 (exemplary for subtype Philadelphia)

The major scope of this work was the transfer of an indirect ELISA used for the “Dresden Panel” into the CL-SMIA principle for subtyping *L. pneumophila* Sg 1. Indirect ELISA assays provide high specificity and sensitivity for *L. pneumophila* typing, allowing rapid screening of a large set of samples. In contrast, the CL-SMIA uses the primary mAbs as cAb. Creating an antibody microarray, a CL image pattern can be generated for each subtype of *L. pneumophila*, as shown in Fig. 1.

A sample is passed over the microarray chip, and the panel of cAbs captures each subtype of *L. pneumophila* Sg 1 at other positions. Due to the structural diversity of the LPS sequences among the different *L. pneumophila* subtypes, simultaneous detection and subtyping are enabled by pattern recognition of each CL microarray image. For this purpose, a biotinylated polyclonal pAb is needed as the primary antibody, which is able to bind specifically to all *L. pneumophila* Sg 1 without cross-reactions to other *Legionella* species, other Sgs, or the immobilized cAb [25]. The flow-based CL-SMIA process is performed on the automated microarray analysis platform MCR-R. The digital CL values of each cAb can be converted into a normalized CL signal. Subtypes of *L. pneumophila* Sg 1 can be easily identified visually or through automated data processing using a ternary code system.

### Evaluation of antibodies for the CL-SMIA principle by ELISA

#### Polyclonal detection antibody

The analysis of detection antibodies is a crucial process to evaluate the specificity and sensitivity of antibody-based assays. The characterization process helps to determine whether antibodies can specifically bind to target antigens, here *Legionella* cells, or bind to other accompanying bacteria in the sample. Hence, the first step was to select for a detection antibody that is specific to all target antigens of *L. pneumophila*, generating a high specificity and sensitivity for the “LegioTyper Panel”. Three commercially available polyclonal antibodies (pAb) were chosen, which bind to at least all *L. pneumophila* Sg and potentially also to other *Legionella* spp. Therefore, 109 non-*Legionella* strains were analyzed, representing the outgroup including all reference strains needed for the validation according to ISO/TS 12869 (see Table 1 for suppl. information). Ninety strains showed no reactivity with none of the pAb, and in contrast, 19 strains showed very low reactivity. As a potential ingroup, all *Legionella* spp. strains were assigned, and the fixed ingroup is *L. pneumophila* Sg 1. In total, 45 *Legionella* species were tested, of which six strains did not bind to the pAb, and 24 strains showed very low reactivity. Nine and five strains bound moderately or respectively strongly to the pAb, among them the *L. longbeachae* and *L. pneumophila* strains. Furthermore, the *L. pneumophila* Sg were tested, and all subgroups of Sg 1 bound well to the pAbs. Additionally, Sg 6 was detected by pAb B65051B and OBT0943, whereas Sg 7 was detected by RD2188412. The remaining serogroups showed low or no binding. For this reason, the biotin-labeled pAb B65051B is the best-suited and most promising detection antibody for its utilization in the CL-SMIA principle.

#### Monoclonal capture antibodies

As known for subtyping *L. pneumophila* Sg 1, the original “Dresden Panel” consists of eight mAb which are used as primary antibody [20]. However, most of the isotypes could not be used as cAb because the immobilization on the microarray chip surface was not possible. Therefore, we screened for alternative cmAb candidates which are applicable for CL-SMIA. We have screened against the mAb library with 242 well-characterized *L. pneumophila* Sg 1 strains, representing the mAb subgroups Knoxville (66 strains), Philadelphia (60 strains), Benidorm (18 strains), France/Allentown (36 strains), OLDA/Oxford (35 strains), Bellingham (11 strains), Heysham (4 strains), and Camperdown (12 strains) (see Table 2 suppl. information). All cmAbs showed a 100% specificity with *L. pneumophila* Sg 1 strains and did not react with other species or serogroups. mAb 81/2 reacted with all *L. pneumophila* Sg 1 isolates by indirect ELISA. The differentiation between isolates of the Pontiac group (mAb 3/1 positive strains in the “Dresden Panel”) and the non-Pontiac group can be facilitated by using the mAb 48/3 and mAb 10/7. Other mAb candidates showed different reactivity patterns compared to the original “Dresden Panel”, but we were able to create a similar decision path for this newly established “LegioTyper Panel”. We replaced mAb 8/4 by mAb 84/2, mAb 26/1 by mAb 30/1, and mAb 30/4 was included to detect the subgroup Camperdown. As a result of tests with indirect ELISA, we determined a set of 9 cmAbs and one cpAb, which we wanted to use as the “Legiotyper Panel” as shown in Table 1.

**Table 1:** Overview of the tested pAb and mAbs (“LegioTyper Panel”) and their binding efficacy to subtypes of *L. pneumophila* Sg1 based on indirect ELISA, usable as cmAb in the CL-SMIA principle

mAb used for the CL-SMIA (“LegioTyper Panel”)	Identified selectivity subtypes of* L. pneumophila* Sg1
pAb	Sg 1
mAb 81/2	Sg 1
mAb 48/3	Pontiac group
mAb 10/7	Non-Pontiac group
mAb 20/1	Benidorm, Bellingham
mAb 3	Knoxville, Heysham
mAb 10/6	Bellingham
mAb 84/2	Philadelphia, OLDA
mAb 30/1	OLDA
mAb 30/4	Benidorm, Heysham, Bellingham

### Transfer of the indirect ELISA to CL-SMIA

**Fig. 2:**
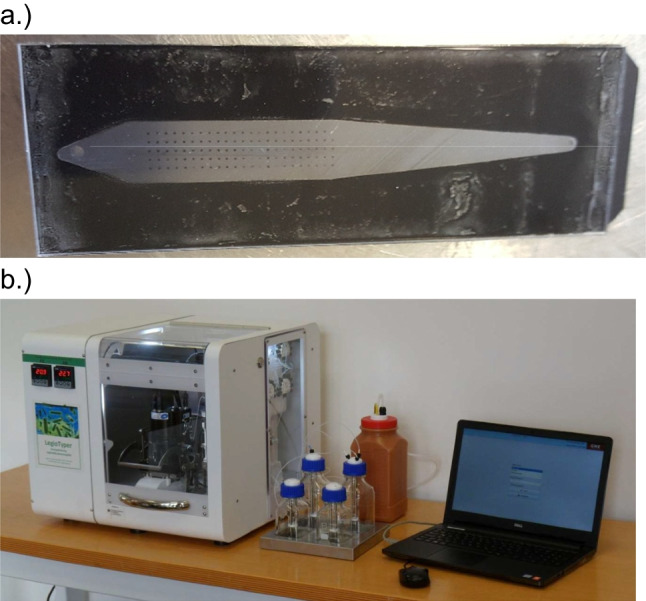
Jeffamine-2003-coated polycarbonate microarray chips (**a**) used for the Legiotyper instrument (**b**) to perform automatically CL-SMIA

The LegioTyper principle is based on an automated flow-based CL-SMIA process on the microarray analysis platform MCR-R to subtype *L. pneumophila* Sg 1 with Jeffamine-2003-coated polycarbonate microarrays (see Fig. 2a). The MCR-R (see Fig. 2b) was a further development of the MCR 3 and is called for this application the LegioTyper instrument.

**Fig. 3:**
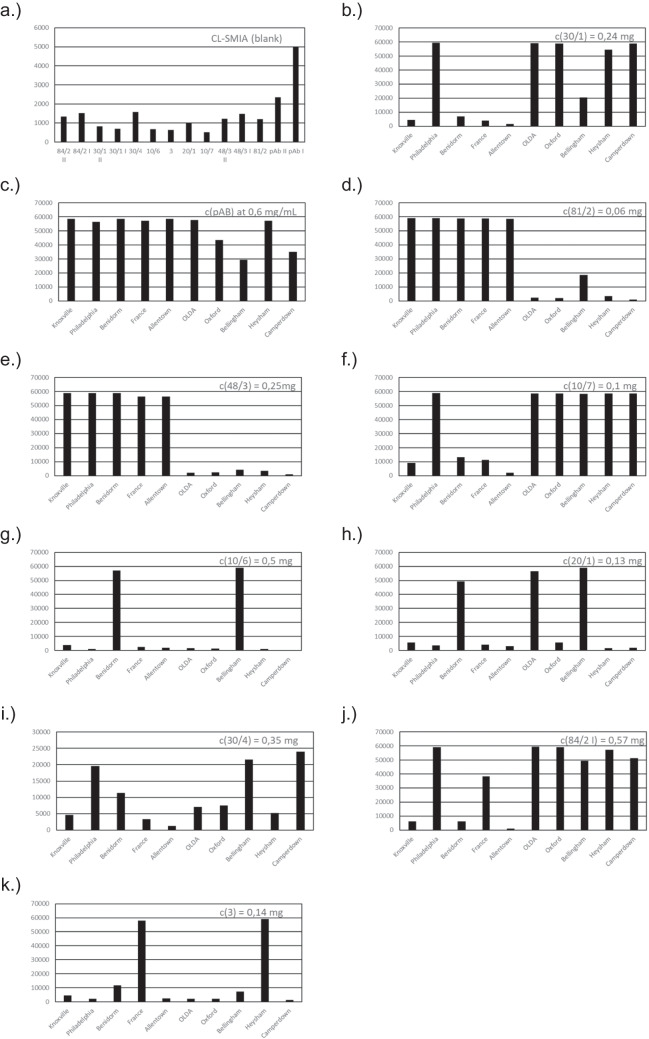
CL intensities of blank measurement (a) and for defined concentration of cAb (b-k) in respect to each subtype of *L*. *pneumophila* Sg1

The usage of this type of plastic instead of glass microarray chips is much simpler and not so expensive to produce compared to glass slides, as described elsewhere in detail [24]. Low costs for LegioTyper chips are important because, applying CL-SMIA for each measurement, a new chip has to be used. Before the “LegioTyper Panel” is tested for applicability, we have programmed the automated CL-SMIA process on MCR-R. The LegioTyper program was optimized regarding low false positive CL signals for each spot of the CL image and rapid testing. The final CL-SMIA process takes 34 min. CL-SMIA with water as a blank showed signals for each cAb with CL signals between 500 a.u. and 5000 a.u., as shown in Fig. 3a. The normalized CL signals in respect to the positive control were between 1 and 8 (highest for cpAb). The reactivity of all cAbs was tested by CL-SMIA with heat inactivated subtypes of *L. pneumophila* Sg1. In comparison with the indirect ELISA, the reactivity of each subtype differed because of the nature of the test methods, such as flow-based approach vs. static incubation and sandwich vs. indirect principle, which includes the necessity of cAb and primary detection pAb. Additionally, the applied antibody concentration in indirect ELISA and CL-SMIA is different. All ten *L. pneumophila* Sg 1 subtypes in a concentration of 10^7^ cfu mL^−1^ were analyzed by the panel of nine cmAbs and one cpAb. Several concentrations of each cAb were tested (data not shown) regarding significantly high CL signals (more than 65% for 100 × (CL_cAb_/CL_max_)). CL_max_ was calculated with 64,157 a.u. by subtracting the mean of CL images without CL reaction (= 1379 a.u for *n* = 10) from the maximal number of the CCD camera (= 65,636 a.u.). Concentrations of cAbs were selected as shown in Fig. 3b–k that subtyping is possible by pattern recognition without overexposure to high CL signals. For cmAb 84/2, 30/1, and 84/3 and cpAb, a high (I) and a low (II) concentration is microcontact printed with the aim that all 10 outbreak-associated subtypes can be separately identified.

Therefore, CL signal intensities were converted into a ternary code using the maximal CL signal CL_max_ for normalization (CL level 0: < 20% of CL_max_; CL level 1: 20%–64% of CL_max_; CL level 2: > 65% of CL_max_).

**Table 2:**
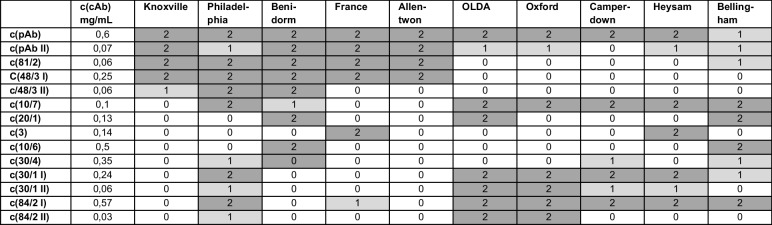
Chosen cAb concentration and the corresponding CL levels for subtypes of *L. pneumophila* Sg1

The concentration of each cAb was selected regarding the highest CL signal for a defined set of subtypes of *L. pneumophila* Sg 1 and low CL signal for the other subtypes. In this way, we could generate a recognition pattern regarding applied cmAbs and cpAb for subtyping *L. pneumophila* Sg 1 (see Table 2). Allentown and Knoxville could be differentiated by using c(48/3 II), which was only detectable with middle CL signal levels (normalized CL signal 20–64% = 1). Subtype France showed high CL signals (normalized CL signal > 65% = 2) for cmAb 3 and CL signal level 1 for cmAb 84/2 I. In contrast to the results of indirect ELISA, cmAb 81/2 bound only to cells of the Pontiac group and was additionally reactive in level 1 for Bellingham. Besides the non-Pontiac group, cmAb 10/7 was highly reactive for Philadelphia and slightly for Benidorm. Not only OLDA was detected by cmAb 30/1 but also the other subtypes of the non-Pontiac group and Philadelphia. Different from the indirect ELISA method, Bellingham and also Benidorm were detected by cmAb 10/6. The reactivity of cmAb 30/4 was only in level 1 for Philadelphia, Bellingham, and Camperdown. OLDA was detected by cmAb 20/1 in addition to Bellingham and Benidorm. Heysham and France, instead of Knoxville, result in high CL signals. Philadelphia and France are highly reactive for cmAb 84/2 and additionally for the non-Pontiac group. Only pAb (all Sg1) and cmAb 48/3 (only Pontiac group) have shown similar results compared to indirect ELISA experiments. In summary, the results of the indirect ELISA and CL-SMIA were not comparable, probably due to an altered topological orientation of mAbs. Therefore, more experiments with more samples and free LPS antigens were conducted with the aim of creating a specific ternary code for each *L. pneumophila* Sg 1 subtype, which is the basis of a decision path comparable to the “Dresden Panel”.

### Creation of a specific ternary code for each* L. pneumophila *Sg 1 subtype

**Fig. 4:**
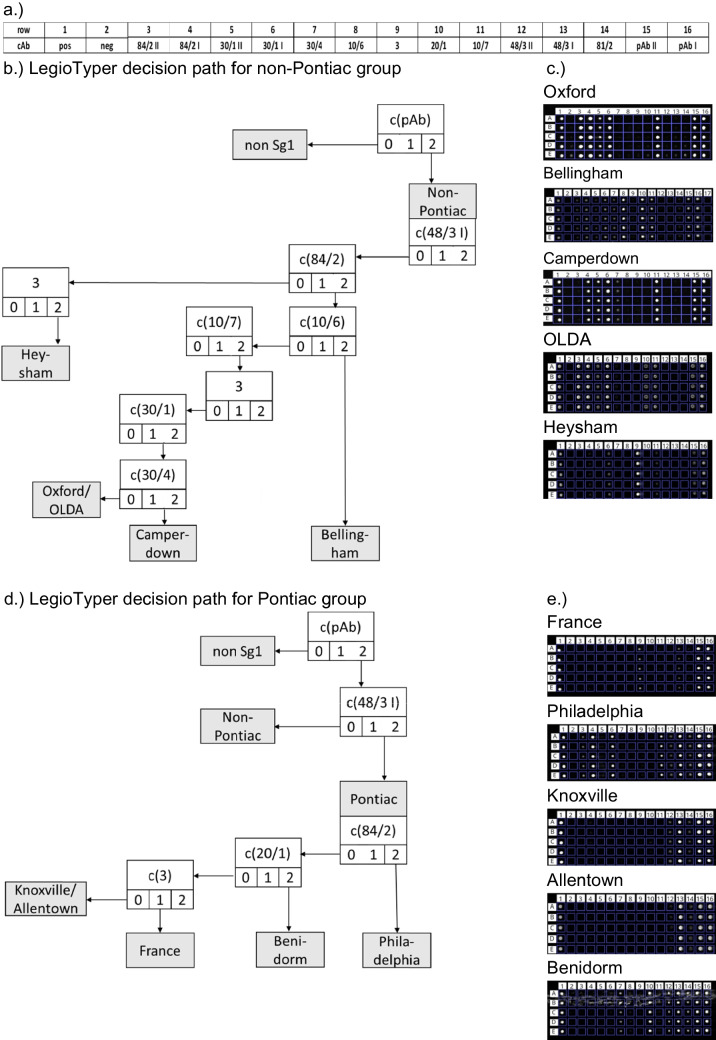
**a** Spotting position of the cAb on the LegioTyper chip; decision path for Pontiac (**b**) and non-Pontiac group (**d**). The start of the outgoing arrows depicts the number of the CL level (0, 1, 2); **c** and **e** examples of CL images of the *L. pneumophila* Sg 1 subtypes

To create the LegioTyper-based decision path that is able to identify subtypes of *L. pneumophila* Sg 1, each subtype was tested with cultivated samples and free LPS antigens. In total, 42 CL-SMIA were conducted on LegioTyper chips. All CL images were processed and evaluated with the ternary code as shown in Table 3. With this huge set of experiments, it was clear that the mean CL signals of the positive control spot (CL_pos_) are the better choice for normalization instead of CL_max_ (CL level 0: < 20% of CL_pos_; CL level 1: 20%–64% of CL_pos_; CL level 2: > 65% of CL_pos_). The maximal CL signals can vary because of various reasons, like different streptavidin-HRP conjugate concentrations or enzyme activities filled in the plastic syringe of the MCR-R. Overall, the normalization by CL_pos_ was successful for every CL-SMIA. The experiments have shown that two different cAb concentrations are not helpful for the subtyping because of too high uncertainties. However, three CL levels 0–1–2 defined from normalized CL values were important to create the LegioTyper decision path (see Fig. 4). Some antigen/antibody combinations did not use strict CL levels but allowed two levels, e.g., CL level 0 and 1 for subtype Philadelphia with mAb 48/3 for finding a decision for the cAb reactivity evaluation. The used cmAbs for each subtype are shown in grey fields. Alternatively, the individual ternary code of Table 3 can be used for digital subtyping in the future. In summary, the new “LegioTyper Panel” is 100% specific for *L. pneumophila* Sg1 and can differentiate between Pontiac and non-Pontiac by cmAb 48/3, as shown for 42 strains. Subtyping was possible for the subtypes Benidorm, Philadelphia, and France from the Pontiac group and for Bellingham, Camperdown, and Heysham as subtypes of the non-Pontiac group. The differentiation between OLDA and Oxford could be performed by cmAb 20/1 because OLDA was identified for 2 from 6 samples with CL level 1 or 2, and for Oxford was only one time measured with CL level 1. However, cmAb 20/1 has shown too high uncertainties and was not used for the decision path. One false negative result was recognized for cmAb 30/4 identifying Oxford instead of Camperdown. The results from cmAb 81/2 and 84/2 were not reproducible for Allentown and Knoxville. Therefore, we decided not to differentiate between these two subtypes. France could be identified by cmAb 3 with a CL level of 1, which was positive for cultured bacteria but not for free LPS antigens. The difficulties in the differentiation of Allentown, France, and Knoxville are similar to the “Dresden Panel”, which is also not able to differentiate between Allentown and France.

**Table 3:**
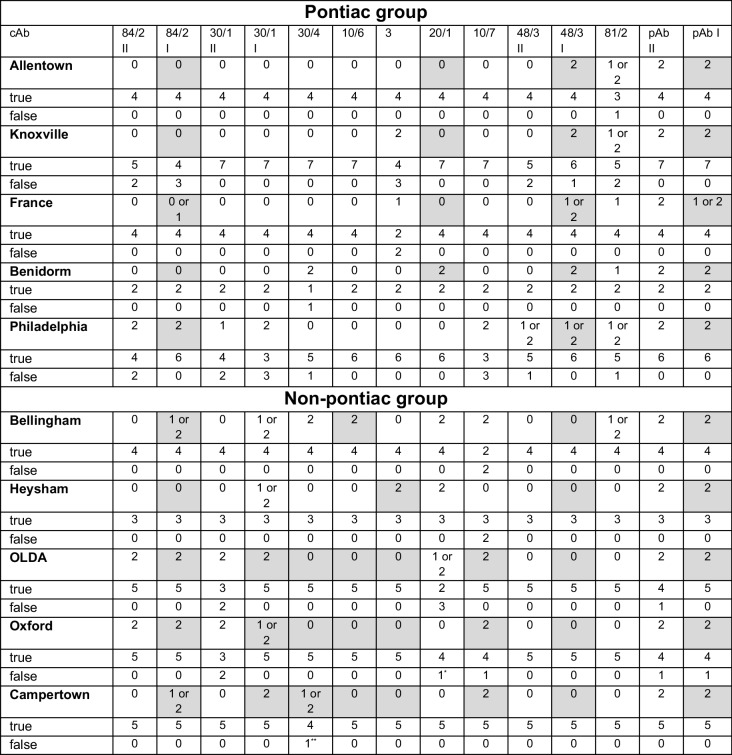
Ternary code specific for subtypes of Pontiac and non-Pontiac group of *L. pneumophila* Sg1

*OLDA

**Oxford or OLDA

The LegioTyper decision path is a simple method to interpret visually each CL image pattern, as shown in Fig. 4b for the Pontiac group and Fig. 4d for the non-Pontiac group. The starting point is the cpAb in position 16 of the microarray image. Next is position 13 with cAb 48/3 I. The spots are level 0 for the non-Pontiac group and either level 1 or 2 for the Pontiac group. Position 14 with cAb 81/2 could not be used for the decision path because the results varied too much. Figure 4c and e show CL images of each *L. pneumophila* Sg 1 subtype which can be used for serotyping. However, a digital evaluation of CL images will be much simpler and more effective in the future using the CL microarray image evaluation software. Finally, we tested seven 10 × concentrated urine samples, of which 5 × were positive for Philadelphia, 1 × for OLDA, and 1 × for France/Allentown using the indirect ELISA of the “Dresden Panel”. For all 6 samples (5 × Philadelphia and 1 × France/Allentown), the cpAb and cmAb 48/3 I were found at CL level 2, identifying Sg1 and the Pontiac group. cmAb 84/2 showed only a slight increase (normalized CL signal 11 ± 6), so the sensitivity has to be increased further to identify Philadelphia in patients’ urine samples or the samples have to be concentrated to a factor of more than 10. The sensitivity of CL-SMIA can be improved by optimizing the concentration of capture antibodies, controlling access to the antibody’s binding site (epitope accessibility) with alternative immobilization strategies, ensuring antibodies are oriented correctly for antigen binding, and using shielding layers to improve the surface-to-volume ratio. These methods aim to increase the number of active, functional antibodies on the microarray surface, leading to a stronger signal and lower detection limits.

## Conclusion and outlook

In this study, we demonstrated that the newly developed “LegioTyper Panel” for rapid subtyping of *L. pneumophila* with cAbs can be used for pattern recognition of CL microarray images after performing a flow-based CL-SMIA. Rapid testing and antibody subtyping of Sg 1 can be performed on the automated CL microarray analysis platform MCR-R within 34 min. The CL image pattern can be interpreted by a decision path similar to the well-known “Dresden Panel”, which is typically performed via indirect ELISA. However, the specificities to the outbreak-associated subtypes of *L. pneumophila* were not comparable. Therefore, concentrations of cAbs for flow-based CL-SMIA were set regarding high CL levels (2) for high binding capacities for a part of cAb. However, some subtypes have shown only middle CL levels (1) or low CL levels (0) in respect to the immobilized cAbs. In this way, a ternary code with cAbs was created for each subtype. In conclusion, the LegioTyper test can be performed with cultivated *Legionella* spp. colonies without further specification of the serogroup by qPCR or agglutination. The ternary code is the basis for digital data evaluation. For visual CL image analysis, we could show that subtyping according to the panel decision paths is applicable for high concentrations of *L. pneumophila* Sg 1 and can successfully be carried out after cultivation. In the field of drinking water regulation, which has stricter requirements than process water monitoring, the German highest public health authority strongly encourages the investigation of *L. pneumophila* isolates mAb 3/1 as the majority of cases and even follow-up cases were linked to drinking water distribution systems that harbor mAb 3/1^+^ strains [25, 26]. This antibody generates positive results associated with the Pontiac subgroup using ELISA, but it was not able to immobilize this antibody cmAb. Therefore, we substituted mAb 3/1 using mAb 48/3 and added mAb 10/7; wherefore, an allocation to *L. pneumophila* Sg 1, the Pontiac, and Non-Pontiac was always 100% able after cultivation. Using the CL-SMIA, all tests can be done with one principle, enabling the comparability between the different samples for risk assessment. Thus, in case of an outbreak, affected patients can be inspected for the responsible subtype, and those results can be compared with environmental culture samples with the similar assay to find rapidly the outbreak source. In such cases, the CL-SMIA can be performed as a rapid test for increasing public health surveillance and outbreak prevention. Rapid tests for *Legionella* detection are gaining increasing importance. Methods such as PCR are now routinely used for the confirmation and typing of *Legionella *spp., *L. pneumophila*, and *L. pneumophila* Sg 1 with faster analysis time and increasing specificity in comparison to culture-based methods [27]. In the future, antibody-based methods like the CL-SMIA could also be introduced as rapid tests for generating the same kind of information with additional subtyping and Sg distinguishing by adding further specific capture and detection antibodies.

## Supplementary Information

Below is the link to the electronic supplementary material.

Supplementary Material 1 (DOCX 25.0 KB)

Supplementary Material 2 (DOCX 38.9 KB)

## Data Availability

Data will be made available upon reasonable request.
