# Supplementary material for: LegioTyper: Rapid typing of Legionella pneumophila serogroup 1 by flow-based chemiluminescence sandwich microarray immunoassay
Source: Anal Bioanal Chem. 2025 Nov 5;417(30):6797–809. doi: 10.1007/s00216-025-06194-3 (PMC12680792; doi:10.1007/s00216-025-06194-3)
Supplement: Supplementary file 1 — Supplementary Material 1 (DOCX 25.0 KB) [file 216_2025_6194_MOESM1_ESM.docx]

LegioTyper: Rapid typing of Legionella pneumophila serogroup 1 by flow-based chemiluminescence sandwich microarray immunoassays

C. Bärwinkel^1^, A. Petzold^2,^ C. Lück^2^, M. Petzold^2*,^ M. Seidel^1*^

^1^Chair of Analytical Chemistry and Water Chemistry, School of Natural Sciences, Technical University of Munich, Lichtenbergstraße 4, 85748 Garching, Bavaria, Germany

^2^Institute of Medical Microbiology and Virology, University Hospital Carl Gustav Carus, Medical Faculty, Dresden University of Technology, Fiedlerstr. 42, 01307 Dresden, Germany

* Shared Corresponding author

Email: michael.seidel@tum.de

Email: Markus.Petzold@ukdd.de

Supplemented Information – Table 1: tested polyclonal antibodies against Legionella and non-Legionella strains by indirect ELISA

|  |  | **reactivity with pAb** | | |
| --- | --- | --- | --- | --- |
| **Species** | **Strain collection No.** | **B65051B** | **OBT0943** | **RD2188412** |
| ***Acidovorax temperans*** | * | neg | neg | neg |
| ***Acinetobacter baumanii*** | DSM 30007 | neg | neg | neg |
| ***Acinetobacter junii*** | DSM 1532 | neg | neg | neg |
| ***Acinetobacter lwoffi*** | DSM 2403 | neg | neg | neg |
| ***Trueperella pyogenes*** | * | neg | neg | neg |
| ***Aerocoocus viridans*** | ATCC 700406 | neg | neg | neg |
| ***Aeromonas eucrenophila*** | * | neg | neg | neg |
| ***Aeromonas hydrophila*** | DSM 6173 | neg | neg | neg |
| ***Aeromonas popoffii*** | * | neg | neg | neg |
| ***Aeromonas sobria*** | * | neg | neg | neg |
| ***Aeromonas veronii*** | DSM 7386 | neg | neg | neg |
| ***Alcaligenes faecalis*** | DSM 6174 | neg | neg | neg |
| ***Arthrobacter pascens*** | * | neg | neg | neg |
| ***Bacillus cereus*** | DSM 345/4490 | + | + | + |
| ***Bacillus licheniformis*** | * | + | + | + |
| ***Bacillus pumilus*** | DSM 27 | + | + | + |
| ***Bacillus subtilis*** | DSM 347 | neg | neg | neg |
| ***Bordatella bronchiseptica*** | DSM 13414 | + | + | + |
| ***Brevibacillus borstelensis*** | * | neg | neg | neg |
| ***Brevibacillus formosus*** | * | neg | neg | neg |
| ***Brevundimonas diminuta*** | DSM 1635 | neg | neg | neg |
| ***Burkholderia cepacia*** | DSM 50180 | neg | neg | neg |
| ***Burkholderia xenovorans*** | * | neg | neg | neg |
| ***Campylobacter jejunii*** | ATCC 33291 | neg | neg | neg |
| ***Citrobacter freudii*** | DSM 30039 | neg | neg | neg |
| ***Citrobacter koseri*** | DSM 4570 | neg | neg | neg |
| ***Claudimonas hydrothermale*** | * | neg | neg | neg |
| ***Clostridiodes difficile*** | DSM 1296 | neg | neg | neg |
| ***Clostridum perfringens*** | DSM 756 | neg | neg | neg |
| ***Clostridum sordelli*** | * | neg | neg | neg |
| ***Corynebacterium callunae*** | DSM 20147 | neg | neg | neg |
| ***Cupriavidus gilardii*** | DSM 17292 | neg | neg | neg |
| ***Enterobacter asburiae*** | * | neg | neg | neg |
| ***Enterobacter cloacae*** | DSM 30054 | neg | neg | neg |
| ***Enterococcus casseliflavus*** | LMG 960 | neg | neg | neg |
| ***Enterococcus durans*** | ATCC 6056 | neg | neg | neg |
| ***Enterococcus faecalis*** | DSM 2570 | neg | neg | neg |
| ***Enterococcus faecum*** | DSM 6177 | neg | neg | neg |
| ***Enterococcus gallinarium*** | ATCC 700425 | neg | neg | neg |
| ***Enterococcus hirae*** | * | neg | neg | neg |
| ***Escherichia coli*** | DSM 30083 | neg | neg | neg |
| ***Exigobacterium auranticum*** | * | neg | neg | neg |
| ***Flavobacterium hibernum*** | DSM 12611 | neg | neg | neg |
| ***Hafnia alvei*** | DSM 30163 | neg | neg | neg |
| ***Haemophilus influenzae*** | DSM 9999 | neg | neg | neg |
| ***Klebsiella aerogenes*** | DSM 30053 | neg | neg | neg |
| ***Klebsiella oxytoca*** | DSM 5175 | neg | neg | neg |
| ***Klebsiella pneumoniae*** | DSM 30104 | neg | neg | neg |
| ***Lelliotta annoigena*** | * | neg | neg | neg |
| ***Leptotrichia trevisanii*** | * | neg | neg | neg |
| ***Limnobacter thiooxidans*** | DSM 13612 | neg | neg | neg |
| ***Listeria grayi*** | DSM 20601 | neg | neg | neg |
| ***Listeria monocytogenes*** | DSM 20600 | neg | neg | neg |
| ***Lysinibacillus sphaericus*** | * | neg | neg | neg |
| ***Microbacterium phyllosphaerae*** | * | neg | neg | neg |
| ***Micrococcus luteus*** | ATCC 700405 | + | + | + |
| ***Moraxella catarrhalis*** | DSM 9143 | neg | neg | neg |
| ***Moraxella osloensis*** | DSM 6998 | neg | neg | neg |
| ***Morganella morganii*** | DSM 30164 | neg | neg | neg |
| ***Mycoplasma pneumoniae*** | DSM 22911 | neg | neg | neg |
| ***Norcadia acidivorans*** | DSM 45049 | + | + | + |
| ***Ochrobactrum anthropi*** | * | neg | neg | neg |
| ***Paenibacillus amylolyticus*** | DSM 15211 | + | + | + |
| ***Paenibacillus polymxa*** | DSM 36 | + | + | + |
| ***Pantoea agglomerans*** | DSM 3493 | neg | neg | neg |
| ***Plesiomonas shigelloides*** | * | neg | neg | neg |
| ***Proteus mirabilis*** | DSM 6674 | neg | neg | neg |
| ***Proteus vulgaris*** | DSM 30118 | neg | neg | neg |
| ***Providencia rettgeri*** | DSM 4542 | + | + | + |
| ***Pseudomonas aeruginosa*** | DSM 50071 | + | + | + |
| ***Pseudomonas cichorii*** | * | neg | neg | neg |
| ***Pseudomonas extremorientalis*** | * | + | + | + |
| ***Pseudomonas fluorescens*** | DSM 50090 | + | + | + |
| ***Pseudomonas grimonti*** | DSM 17515 | neg | neg | neg |
| ***Pseudomonas koreensis*** | DSM 16610 | neg | neg | neg |
| ***Pseudomonas libanensis*** | DSM 17149 | neg | neg | neg |
| ***Pseudomonas putida*** | DSM 291 | + | + | + |
| ***Pseudomonas luteola*** | DSM 6975 | + | + | + |
| ***Pseudomonas rhodesiae*** | DSM 14020 | + | + | + |
| ***Pseudomonas veronii*** | DSM 11331 | + | + | + |
| ***Rahnella aquatilis*** | DSM 4594 | neg | neg | neg |
| ***Salmonella typhimurium*** | NCTC 12023 | neg | neg | neg |
| ***Serratia fonticola*** | DSM 4576 | neg | neg | neg |
| ***Serratia liquefaciens*** | * | neg | neg | neg |
| ***Serratia marcescens*** | DSM 30121 | neg | neg | neg |
| ***Serratia odorifera*** | * | neg | neg | neg |
| ***Serratia plymuthica*** | DSM 4540 | + | + | + |
| ***Shewanella baltica*** | DSM 9439 | + | + | + |
| ***Shigella dysenteriae*** | DSM 4781 | neg | neg | neg |
| ***Solibacterium silvestris*** | * | neg | neg | neg |
| ***Sphingobacterium multivorum*** | DSM 6175 | neg | neg | neg |
| ***Sphingobacterium spiritivorum*** | DSM 2582 | neg | neg | neg |
| ***Sphingomonas koreensis*** | * | neg | neg | neg |
| ***Sphingomonas paucimobilis*** | DSM 1098 | neg | neg | neg |
| ***Staphylococcu aureus*** | DSM2569 | neg | neg | neg |
| ***Staphylococcus aureus*** | DSM 13661 | neg | neg | neg |
| ***Staphylococcus capitis*** | DSM 6180 | + | + | + |
| ***Staphylococcus epidermidis*** | DSM 1798 | neg | neg | neg |
| ***Staphylococcus gallinarum*** | ATCC 700401 | neg | neg | neg |
| ***Staphylococcus lugdunensis*** | ATCC 49576 | neg | neg | neg |
| ***Staphylococcus warneri*** | DSM 20316 | neg | neg | neg |
| ***Staphylocoocus hominis*** | DSM 20328 | neg | neg | neg |
| ***Staphylococcus saprophyticus*** | DSM 20229 | neg | neg | neg |
| ***Stenotrophomonas maltophilia*** | ATCC 51331 | neg | neg | neg |
| ***Streptococcus equi*** | ATCC 9528 | neg | neg | neg |
| ***Streptococcus pyogenes*** | DSM 11728 | neg | neg | neg |
| ***Virgibacillus neidei*** | * | neg | neg | neg |
| ***Yersinia enterolitica*** | DSM 11067 | neg | neg | neg |
| ***Yersinia ruckeri*** | DSM 18506 | neg | neg | neg |
|  |  |  |  |  |
| ***L. adelaidensis*** | ATCC 49625 | + | + | + |
| **L. anisa** | ATCC 35292 | + | + | + |
| ***L. beliardensis*** | ATCC 700512 | ++ | ++ | ++ |
| ***L. birminghamensis*** | DSM 19232 | + | + | + |
| ***L. bozemanae*** | ATCC 33217 | ++ | ++ | ++ |
| ***L. brunensis*** | DSM 19236 | + | + | + |
| ***L. cherrii*** | ATCC 35252 | + | + | + |
| ***L. cincinnatiensis*** | ATCC 43753 | + | + | + |
| ***L. dresdenensis*** | DSM 19488 | + | + | + |
| ***L. dumoffii*** | ATCC 33279 | neg | neg | neg |
| ***L. erythra*** | ATCC 35303 | + | + | + |
| ***L. fairfieldensis*** | NCTC 12488 | + | + | + |
| ***L. feeleiI Sg1*** | NCTC 12022 | + | + | + |
| ***L. feeleii Sg1*** | NCTC 11978 | + | + | + |
| ***L. geestiana*** | DSM 21217 | + | + | + |
| ***L. gormanii*** | ATCC 33297 | + | + | + |
| ***L. gratiana*** | ATCC 49504 | +++ | +++ | +++ |
| ***L. gresilenis*** | DSM 21218 | ++ | ++ | ++ |
| ***L. impletisoli*** | DSM 18493 | + | + | + |
| ***L. israelensis*** | NCTC 12010 | + | + | + |
| ***L. jamestowniensis*** | NCTC 11981 | neg | neg | neg |
| ***L. jordanis*** | ATCC 33623 | neg | neg | neg |
| ***L. londinienis*** | NCTC 12374 | + | + | + |
| ***L. longbeachae Sg1*** | ATCC 33462 | +++ | +++ | +++ |
| ***L. longbeachae Sg2*** | ATCC 33484 | ++ | ++ | ++ |
| ***L. maceachernii*** | ATCC 35300 | + | + | + |
| ***L. nautarum*** | ATCC 49506 | + | + | + |
| ***L. norrlandica*** | DSM 105104 | +++ | +++ | +++ |
| ***L. osnabrueckensis*** | W05-934-2 | + | + | + |
| ***L. parisiensis*** | ATCC 35299 | + | + | + |
| ***L. quateirensis*** | ATCC 49507 | ++ | ++ | ++ |
| **L. quinlivanii** | DSM 21216 | + | + | + |
| ***L. rubrilucens*** | DSM 11884 | neg | neg | neg |
| ***L. sainthelensi*** | ATCC 35248 | ++ | ++ | ++ |
| ***L. santicrucis*** | ATCC 35301 | +++ | +++ | +++ |
| ***L. shakespearei*** | ATCC 49655 | + | + | + |
| ***L. spiritensis*** | ATCC 35249 | + | + | + |
| ***L. steigerwaltii*** | ATCC 35302 | ++ | ++ | ++ |
| ***L. taurinensis*** | ATCC 700508 | neg | neg | neg |
| ***L. tusconensis*** | ATCC 49180 | neg | neg | neg |
| ***L. wadsworthii*** | ATCC 33877 | + | + | + |
| ***L. waltersii*** | DSM 21908 | +++ | +++ | +++ |
| **L. worsleiensis** | NCTC 12377 | ++ | ++ | ++ |
| **L. yabuuchiae** | DSM 18492 | ++ | ++ | ++ |
| **L. pneumophila SG 1 - Philadelphia** | ATCC 33152 | +++ | +++ | +++ |
| **L. pneumophila SG 1 - Heysham** | ATCC 43107 | +++ | +++ | +++ |
| **L. pneumophila SG 1 - Camperdown** | ATCC 43113 | +++ | +++ | +++ |
| **L. pneumophila SG 1 - Knxoville** | NCTC 11286 | +++ | +++ | +++ |
| **L. pneumophila SG 1 - OLDA** | NCTC 12008 | +++ | +++ | +++ |
| **L. pneumophila SG 1 - Benidorm** | NCTC 12006 | +++ | +++ | +++ |
| **L. pneumophila SG 1 - Allentown** | NCTC 12024 | +++ | +++ | +++ |
| **L. pneumophila SG 1 - Oxford** | NCTC 12009 | +++ | +++ | +++ |
| **L. pneumophila SG 1 - Bellingham** | NCTC 11404 | +++ | +++ | +++ |
| **L. pneumophila SG 1 - France** | NCTC 12007 | +++ | +++ | +++ |
| **L. pneumophila SG 2** | DSM 25071 | + | + | neg |
| **L. pneumophila SG 3** | DSM 25119 | + | + | neg |
| **L. pneumophila SG 4** | DSM 7514 | + | + | + |
| **L. pneumophila SG 5 - Dallas** | DSM 24991 | + | + | + |
| **L. pneumophila SG 6 - Chicago** | DSM 25182 | +++ | +++ | + |
| **L. pneumophila SG 7** | ATCC 33823 | + | + | +++ |
| **L. pneumophila SG 8** | DSM 25000 | + | neg | + |
| **L. pneumophila SG 9** | ATCC 35289 | + | + | + |
| **L. pneumophila SG 10** | DSM 25061 | + | + | + |
| **L. pneumophila SG 11** | DSM 25063 | + | + | + |
| **L. pneumophila SG 12** | DSM 25224 | + | + | + |
| **L. pneumophila SG 13** | DSM 25225 | neg | neg | + |
| **L. pneumophila SG 14** | DSM 25226 | + | + | + |
| **L. pneumophila SG 15** | ATCC 35251 | + | + | + |
|  |  |  |  |  |
| **** own strain collection: Species assigned by Maldi-TOF score > 2.00 (Bruker Biotyper)*** | | | |  |
