# Supplementary material for: LegioTyper: Rapid typing of Legionella pneumophila serogroup 1 by flow-based chemiluminescence sandwich microarray immunoassay
Source: Anal Bioanal Chem. 2025 Nov 5;417(30):6797–809. doi: 10.1007/s00216-025-06194-3 (PMC12680792; doi:10.1007/s00216-025-06194-3)
Supplement: Supplementary file 2 — Supplementary Material 2 (DOCX 38.9 KB) [file 216_2025_6194_MOESM2_ESM.docx]

LegioTyper: Rapid typing of Legionella pneumophila serogroup 1 by flow-based chemiluminescence sandwich microarray immunoassays

C. Bärwinkel^1^, A. Petzold^2,^ C. Lück^2^, M. Petzold^2*,^ M. Seidel^1*^

^1^Chair of Analytical Chemistry and Water Chemistry, School of Natural Sciences, Technical University of Munich, Lichtenbergstraße 4, 85748 Garching, Bavaria, Germany

^2^Institute of Medical Microbiology and Virology, University Hospital Carl Gustav Carus, Medical Faculty, Dresden University of Technology, Fiedlerstr. 42, 01307 Dresden, Germany

* Shared Corresponding author

Email: michael.seidel@tum.de

Email: [Markus.Petzold@ukdd.de](mailto:Markus.Petzold@ukdd.de)

Supplemented Information – Table 2: tested monoclonal antibodies against strains of *L. pneumophila* Sg 1 by indirect ELISA

| **Strain** | **mAb-subgroup** | **monoclonal antibodies (mAb)** | | | | | | | | | | | | |
| --- | --- | --- | --- | --- | --- | --- | --- | --- | --- | --- | --- | --- | --- | --- |
|  |  | **8/5** | **81/2** | **3/1** | **48/3** | **8/4** | **84/2** | **3** | **10/7** | **30/4** | **10/6** | **20/1** | **26/1** | **30/1** |
| **EUL 13** | Benidorm | +++ | +++ | +++ | +++ | neg | neg | neg | neg | + | neg | +++ | neg | neg |
| **EUL 14** | Benidorm | +++ | +++ | +++ | +++ | neg | neg | neg | neg | neg | neg | +++ | + | + |
| **EUL 16** | Benidorm | +++ | +++ | +++ | +++ | neg | neg | neg | neg | + | neg | +++ | neg | neg |
| **EUL 20-1** | Benidorm | +++ | +++ | +++ | +++ | neg | neg | neg | neg | + | neg | +++ | ++ | ++ |
| **EUL 20-2** | Benidorm | +++ | +++ | +++ | +++ | neg | neg | neg | neg | neg | neg | +++ | neg | neg |
| **EUL 20-1** | Benidorm | +++ | +++ | +++ | +++ | neg | neg | neg | neg | + | neg | +++ | neg | neg |
| **EUL 20-2** | Benidorm | +++ | +++ | +++ | +++ | neg | neg | neg | neg | + | neg | +++ | neg | neg |
| **EUL 27** | Benidorm | +++ | +++ | +++ | +++ | neg | neg | neg | neg | + | neg | +++ | neg | neg |
| **EUL 32** | Benidorm | +++ | +++ | +++ | +++ | neg | neg | neg | neg | + | neg | +++ | + | + |
| **EUL 39** | Benidorm | +++ | +++ | +++ | +++ | neg | neg | neg | neg | + | neg | +++ | + | + |
| **EUL 50** | Benidorm | +++ | +++ | +++ | +++ | neg | neg | neg | neg | neg | neg | +++ | + | + |
| **EUL 68** | Benidorm | +++ | +++ | +++ | +++ | neg | neg | neg | neg | + | neg | +++ | + | + |
| **EUL 75** | Benidorm | +++ | +++ | +++ | +++ | neg | neg | neg | neg | + | neg | +++ | neg | neg |
| **EUL 83** | Benidorm | +++ | +++ | +++ | +++ | neg | neg | neg | neg | + | + | +++ | + | + |
| **EUL 101** | Benidorm | +++ | +++ | +++ | +++ | neg | neg | neg | neg | + | neg | +++ | neg | neg |
| **EUL 105** | Benidorm | +++ | +++ | +++ | +++ | neg | neg | neg | neg | + | neg | +++ | neg | neg |
| **EUL 111** | Benidorm | +++ | +++ | +++ | +++ | neg | neg | neg | neg | + | neg | +++ | neg | neg |
| **EUL 117** | Benidorm | +++ | +++ | +++ | +++ | neg | neg | neg | neg | + | + | +++ | neg | neg |
| **EUL 48-2** | **Bellingham** | +++ | +++ | neg | neg | neg | neg | neg | neg | +++ | +++ | +++ | neg | neg |
| **EUL 81** | **Bellingham** | +++ | +++ | neg | neg | neg | neg | neg | neg | +++ | +++ | +++ | neg | neg |
| **EUL 91** | **Bellingham** | +++ | +++ | neg | neg | neg | neg | neg | neg | +++ | +++ | +++ | + | + |
| **EUL 99** | **Bellingham** | +++ | +++ | neg | neg | neg | neg | neg | neg | +++ | +++ | +++ | + | + |
| **EUL 102** | **Bellingham** | +++ | +++ | neg | neg | neg | neg | neg | neg | +++ | +++ | +++ | + | + |
| **EUL 18** | **Bellingham** | +++ | +++ | neg | neg | neg | neg | neg | neg | +++ | +++ | +++ | + | + |
| **EU 48-2** | **Bellingham** | +++ | +++ | neg | neg | neg | neg | neg | neg | +++ | +++ | +++ | neg | + |
| **EUL 81** | **Bellingham** | +++ | +++ | neg | neg | neg | neg | neg | neg | +++ | +++ | +++ | neg | neg |
| **EUL 91** | **Bellingham** | +++ | +++ | neg | neg | neg | neg | neg | neg | +++ | +++ | +++ | neg | neg |
| **EUL 102** | **Bellingham** | +++ | +++ | neg | neg | neg | neg | neg | neg | +++ | +++ | +++ | neg | neg |
| **EUL 100** | **Bellingham** | +++ | +++ | neg | neg | neg | neg | neg | neg | +++ | +++ | +++ | neg | neg |
| **EUL 2** | **Philadelphia** | +++ | +++ | +++ | +++ | +++ | +++ | neg | + | + | neg | + | + | + |
| **EUL 3** | **Philadelphia** | +++ | +++ | +++ | +++ | +++ | +++ | neg | + | ++ | neg | + | neg | + |
| **EUL 7** | **Philadelphia** | +++ | +++ | +++ | +++ | +++ | +++ | neg | + | ++ | + | + | + | + |
| **EUL 9** | **Philadelphia** | +++ | +++ | +++ | +++ | +++ | +++ | neg | + | ++ | neg | + | ++ | ++ |
| **EUL 10** | **Philadelphia** | +++ | +++ | +++ | +++ | +++ | +++ | neg | + | ++ | neg | + | + | + |
| **EUL 17** | **Philadelphia** | +++ | +++ | +++ | +++ | +++ | +++ | neg | + | ++ | + | + | + | + |
| **EUL 21** | **Philadelphia** | +++ | +++ | +++ | +++ | +++ | +++ | neg | + | ++ | + | + | neg | + |
| **EUL 37** | **Philadelphia** | +++ | +++ | +++ | +++ | +++ | +++ | neg | + | ++ | + | + | + | + |
| **EUL 60** | **Philadelphia** | +++ | +++ | +++ | +++ | +++ | +++ | neg | + | ++ | neg | + | + | + |
| **EUL 43** | **Philadelphia** | +++ | +++ | +++ | +++ | +++ | +++ | + | + | ++ | neg | + | + | + |
| **EUL 69** | **Philadelphia** | +++ | +++ | +++ | +++ | +++ | +++ | + | + | + | neg | + | neg | neg |
| **EUL 74** | **Philadelphia** | +++ | +++ | +++ | +++ | +++ | +++ | + | + | ++ | ++ | + | neg | neg |
| **EUL 118** | **Philadelphia** | +++ | +++ | +++ | +++ | +++ | +++ | + | + | + | + | + | + | + |
| **EUL 52** | **Philadelphia** | +++ | +++ | +++ | +++ | +++ | +++ | + | + | + | + | + | + | + |
| **EUL 60** | **Philadelphia** | +++ | +++ | +++ | +++ | +++ | +++ | + | + | + | neg | + | neg | neg |
| **EUL 43** | **Philadelphia** | +++ | +++ | +++ | +++ | +++ | +++ | + | neg | + | neg | neg | + | + |
| **EUL 41** | **Philadelphia** | +++ | +++ | +++ | +++ | +++ | +++ | + | + | neg | neg | neg | + | + |
| **L01-403** | **Philadelphia** | +++ | +++ | +++ | +++ | +++ | +++ | neg | neg | neg | neg | neg | + | + |
| **L15-235** | **Philadelphia** | +++ | +++ | +++ | +++ | +++ | +++ | neg | + | neg | neg | neg | + | + |
| **L15-249** | **Philadelphia** | +++ | +++ | +++ | +++ | +++ | +++ | neg | + | + | neg | neg | + | + |
| **L11-398** | **Philadelphia** | +++ | +++ | +++ | +++ | +++ | +++ | + | + | neg | neg | neg | neg | + |
| **L1-403** | **Philadelphia** | +++ | +++ | +++ | +++ | +++ | +++ | neg | + | neg | neg | neg | + | + |
| **L14-186** | **Philadelphia** | +++ | +++ | +++ | +++ | +++ | +++ | neg | neg | neg | neg | neg | + | + |
| **L14-380** | **Philadelphia** | +++ | +++ | +++ | +++ | +++ | +++ | neg | + | neg | neg | neg | neg | neg |
| **L15-109** | **Philadelphia** | +++ | +++ | +++ | +++ | +++ | +++ | + | + | neg | neg | neg | neg | neg |
| **L15-141** | **Philadelphia** | +++ | +++ | +++ | +++ | +++ | +++ | + | neg | neg | neg | neg | neg | neg |
| **L15-249** | **Philadelphia** | +++ | +++ | +++ | +++ | +++ | +++ | + | neg | neg | neg | neg | neg | neg |
| **L31-31** | **Philadelphia** | +++ | +++ | +++ | +++ | +++ | +++ | neg | neg | neg | neg | neg | + | + |
| **W04-670** | **Philadelphia** | +++ | +++ | +++ | +++ | +++ | +++ | + | + | neg | neg | neg | neg | neg |
| **W12-11190** | **Philadelphia** | +++ | +++ | +++ | +++ | +++ | +++ | + | neg | neg | neg | neg | + | + |
| **W13-309** | **Philadelphia** | +++ | +++ | +++ | +++ | +++ | +++ | + | neg | neg | neg | neg | neg | neg |
| **L11-154** | **Philadelphia** | +++ | +++ | +++ | +++ | +++ | +++ | + | neg | neg | neg | neg | neg | neg |
| **L13-309** | **Philadelphia** | +++ | +++ | +++ | +++ | +++ | +++ | neg | neg | neg | neg | neg | + | + |
| **L14-186** | **Philadelphia** | +++ | +++ | +++ | +++ | +++ | +++ | + | neg | neg | neg | neg | neg | neg |
| **L15-084** | **Philadelphia** | +++ | +++ | +++ | +++ | +++ | +++ | + | neg | neg | neg | neg | neg | neg |
| **L15-109** | **Philadelphia** | +++ | +++ | +++ | +++ | +++ | +++ | + | neg | neg | neg | neg | neg | neg |
| **L15-124** | **Philadelphia** | +++ | +++ | +++ | +++ | +++ | +++ | + | neg | + | neg | neg | neg | neg |
| **L15-197** | **Philadelphia** | +++ | +++ | +++ | +++ | +++ | +++ | + | neg | neg | neg | neg | neg | neg |
| **L15-286** | **Philadelphia** | +++ | +++ | +++ | +++ | +++ | +++ | neg | neg | neg | neg | neg | neg | neg |
| **L10-232** | **Philadelphia** | +++ | +++ | +++ | +++ | +++ | +++ | + | + | neg | neg | neg | + | + |
| **L16-348** | **Philadelphia** | +++ | +++ | +++ | +++ | +++ | +++ | + | + | neg | neg | + | neg | neg |
| **L16-342** | **Philadelphia** | +++ | +++ | +++ | +++ | +++ | +++ | + | neg | neg | neg | + | neg | neg |
| **L31-31** | **Philadelphia** | +++ | +++ | +++ | +++ | +++ | +++ | + | + | neg | neg | neg | neg | neg |
| **L11-154** | **Philadelphia** | +++ | +++ | +++ | +++ | +++ | +++ | + | neg | neg | neg | neg | neg | neg |
| **L11-398** | **Philadelphia** | +++ | +++ | +++ | +++ | +++ | +++ | + | neg | neg | neg | neg | neg | neg |
| **L1-154** | **Philadelphia** | +++ | +++ | +++ | +++ | +++ | +++ | + | neg | neg | neg | neg | neg | neg |
| **L13-309** | **Philadelphia** | +++ | +++ | +++ | +++ | +++ | +++ | + | neg | neg | neg | neg | neg | neg |
| **L1-403** | **Philadelphia** | +++ | +++ | +++ | +++ | +++ | +++ | + | neg | neg | neg | neg | neg | neg |
| **L14-186** | **Philadelphia** | +++ | +++ | +++ | +++ | +++ | +++ | + | neg | neg | neg | neg | neg | neg |
| **L15-084** | **Philadelphia** | +++ | +++ | +++ | +++ | +++ | +++ | + | neg | neg | neg | neg | neg | neg |
| **L15-109** | **Philadelphia** | +++ | +++ | +++ | +++ | +++ | +++ | + | neg | neg | neg | neg | neg | neg |
| **L15-124** | **Philadelphia** | +++ | +++ | +++ | +++ | +++ | +++ | + | neg | neg | neg | neg | neg | neg |
| **L15-141** | **Philadelphia** | +++ | +++ | +++ | +++ | +++ | +++ | + | neg | neg | neg | neg | neg | neg |
| **L15-197** | **Philadelphia** | +++ | +++ | +++ | +++ | +++ | +++ | + | neg | + | neg | neg | neg | neg |
| **L15-249** | **Philadelphia** | +++ | +++ | +++ | +++ | +++ | +++ | + | + | neg | neg | neg | + | + |
| **L15-286** | **Philadelphia** | +++ | +++ | +++ | +++ | +++ | +++ | neg | + | neg | neg | neg | neg | + |
| **L16-348** | **Philadelphia** | +++ | +++ | +++ | +++ | +++ | +++ | + | neg | neg | neg | neg | neg | neg |
| **L31-31** | **Philadelphia** | +++ | +++ | +++ | +++ | +++ | +++ | + | neg | neg | neg | neg | neg | neg |
| **L16-200** | **Philadelphia** | +++ | +++ | +++ | +++ | +++ | +++ | + | neg | neg | neg | + | neg | neg |
| **L16-200** | **Philadelphia** | +++ | +++ | +++ | +++ | +++ | +++ | + | neg | neg | neg | + | neg | neg |
| **EUL 38-1** | **OLDA/Oxford** | +++ | +++ | neg | neg | +++ | +++ | neg | +++ | ++ | neg | neg | +++ | +++ |
| **EUL 103** | **OLDA/Oxford** | +++ | +++ | neg | neg | +++ | +++ | neg | +++ | ++ | neg | neg | +++ | +++ |
| **EUL 104** | **OLDA/Oxford** | +++ | +++ | neg | neg | +++ | +++ | neg | +++ | ++ | neg | neg | +++ | +++ |
| **EUL 110** | **OLDA/Oxford** | +++ | +++ | neg | neg | +++ | +++ | neg | +++ | ++ | neg | neg | +++ | +++ |
| **EUL 116-3** | **OLDA/Oxford** | +++ | +++ | neg | neg | +++ | +++ | neg | +++ | ++ | neg | neg | +++ | +++ |
| **EUL 53** | **OLDA/Oxford** | +++ | +++ | neg | neg | +++ | +++ | neg | +++ | ++ | neg | neg | +++ | +++ |
| **EUL 55** | **OLDA/Oxford** | +++ | +++ | neg | neg | +++ | +++ | neg | +++ | ++ | neg | neg | +++ | +++ |
| **EUL 84** | **OLDA/Oxford** | +++ | +++ | neg | neg | +++ | +++ | neg | +++ | ++ | neg | neg | +++ | +++ |
| **EUL 88** | **OLDA/Oxford** | +++ | +++ | neg | neg | +++ | +++ | neg | +++ | ++ | neg | neg | +++ | +++ |
| **WL 211-28** | **OLDA/Oxford** | +++ | +++ | neg | neg | +++ | +++ | neg | +++ | ++ | neg | neg | +++ | +++ |
| **WL 211-49** | **OLDA/Oxford** | +++ | +++ | neg | neg | +++ | +++ | neg | +++ | ++ | neg | neg | +++ | +++ |
| **WL 211-50** | **OLDA/Oxford** | +++ | +++ | neg | neg | +++ | +++ | neg | +++ | ++ | neg | neg | +++ | +++ |
| **WL 211-51** | **OLDA/Oxford** | +++ | +++ | neg | neg | +++ | +++ | neg | +++ | ++ | neg | neg | +++ | +++ |
| **WL 211-53** | **OLDA/Oxford** | +++ | +++ | neg | neg | +++ | +++ | neg | +++ | ++ | neg | neg | +++ | +++ |
| **WL 219-36** | **OLDA/Oxford** | +++ | +++ | neg | neg | +++ | +++ | neg | +++ | ++ | neg | neg | +++ | +++ |
| **L10-226** | **OLDA/Oxford** | +++ | +++ | neg | neg | +++ | +++ | neg | +++ | ++ | neg | neg | +++ | +++ |
| **L15-123** | **OLDA/Oxford** | +++ | +++ | neg | neg | +++ | +++ | neg | +++ | ++ | neg | neg | +++ | +++ |
| **W03-610** | **OLDA/Oxford** | +++ | +++ | neg | neg | +++ | +++ | neg | +++ | ++ | neg | neg | +++ | +++ |
| **W10-46** | **OLDA/Oxford** | +++ | +++ | neg | neg | +++ | +++ | neg | +++ | ++ | neg | neg | +++ | +++ |
| **W10-52** | **OLDA/Oxford** | +++ | +++ | neg | neg | +++ | +++ | neg | +++ | ++ | neg | neg | +++ | +++ |
| **W11-929** | **OLDA/Oxford** | +++ | +++ | neg | neg | +++ | +++ | neg | +++ | ++ | neg | neg | +++ | +++ |
| **W14-890** | **OLDA/Oxford** | +++ | +++ | neg | neg | +++ | +++ | neg | +++ | ++ | neg | neg | +++ | +++ |
| **W16-399-5** | **OLDA/Oxford** | +++ | +++ | neg | neg | +++ | +++ | neg | +++ | ++ | neg | neg | +++ | +++ |
| **W16-400-5** | **OLDA/Oxford** | +++ | +++ | neg | neg | +++ | +++ | neg | +++ | ++ | neg | neg | +++ | +++ |
| **W16-403-3** | **OLDA/Oxford** | +++ | +++ | neg | neg | +++ | +++ | neg | +++ | ++ | neg | neg | +++ | +++ |
| **W16-425** | **OLDA/Oxford** | +++ | +++ | neg | neg | +++ | +++ | neg | +++ | ++ | + | neg | +++ | +++ |
| **W16-429** | **OLDA/Oxford** | +++ | +++ | neg | neg | +++ | +++ | neg | +++ | ++ | neg | neg | +++ | +++ |
| **W16-430** | **OLDA/Oxford** | +++ | +++ | neg | neg | +++ | +++ | neg | +++ | ++ | neg | neg | +++ | +++ |
| **W16-431** | **OLDA/Oxford** | +++ | +++ | neg | neg | +++ | +++ | neg | +++ | ++ | neg | neg | +++ | +++ |
| **W16-892** | **OLDA/Oxford** | +++ | +++ | neg | neg | +++ | +++ | neg | +++ | ++ | neg | neg | +++ | +++ |
| **WL191-3** | **OLDA/Oxford** | +++ | +++ | neg | neg | +++ | +++ | neg | +++ | ++ | neg | neg | +++ | +++ |
| **WL196-6** | **OLDA/Oxford** | +++ | +++ | neg | neg | +++ | +++ | neg | +++ | ++ | neg | neg | +++ | +++ |
| **L16-342** | **OLDA/Oxford** | +++ | +++ | neg | neg | +++ | +++ | neg | +++ | ++ | neg | neg | +++ | +++ |
| **L10-232** | **OLDA/Oxford** | +++ | +++ | neg | neg | +++ | +++ | neg | +++ | ++ | neg | neg | +++ | +++ |
| **W03-102** | **OLDA/Oxford** | +++ | +++ | neg | neg | +++ | +++ | neg | +++ | ++ | neg | neg | +++ | +++ |
| **L15-171** | **France/Allentown** | +++ | +++ | +++ | +++ | + | + | neg | + | neg | neg | neg | + | + |
| **WL 332** | **France/Allentown** | +++ | +++ | +++ | +++ | + | + | neg | + | neg | neg | neg | + | + |
| **WL 334** | **France/Allentown** | +++ | +++ | +++ | +++ | neg | neg | neg | neg | neg | neg | neg | neg | neg |
| **WL 334** | **France/Allentown** | +++ | +++ | +++ | +++ | neg | neg | neg | neg | neg | neg | neg | neg | neg |
| **WL 211-34** | **France/Allentown** | +++ | +++ | +++ | +++ | neg | neg | neg | neg | neg | neg | neg | neg | neg |
| **WL 213-19** | **France/Allentown** | +++ | +++ | +++ | +++ | neg | neg | neg | + | neg | neg | + | + | + |
| **WL 219-49** | **France/Allentown** | +++ | +++ | +++ | +++ | neg | neg | neg | neg | neg | + | neg | + | + |
| **L01-520** | **France/Allentown** | +++ | +++ | +++ | +++ | neg | neg | neg | neg | neg | neg | neg | + | + |
| **L03-316** | **France/Allentown** | +++ | +++ | +++ | +++ | neg | neg | neg | + | neg | neg | neg | + | + |
| **L09-346** | **France/Allentown** | +++ | +++ | +++ | +++ | neg | neg | neg | + | neg | neg | neg | neg | + |
| **L12-360** | **France/Allentown** | +++ | +++ | +++ | +++ | neg | neg | neg | + | neg | neg | neg | neg | neg |
| **L13-207** | **France/Allentown** | +++ | +++ | +++ | +++ | neg | neg | neg | + | neg | neg | neg | neg | neg |
| **L13-429** | **France/Allentown** | +++ | +++ | +++ | +++ | neg | neg | neg | + | neg | neg | neg | + | + |
| **L13-596** | **France/Allentown** | +++ | +++ | +++ | +++ | neg | neg | neg | + | neg | neg | neg | + | + |
| **L14-001** | **France/Allentown** | +++ | +++ | +++ | +++ | neg | neg | neg | + | neg | neg | neg | + | + |
| **L14-155** | **France/Allentown** | +++ | +++ | +++ | +++ | neg | neg | neg | + | neg | neg | neg | + | + |
| **L15-040** | **France/Allentown** | +++ | +++ | +++ | +++ | neg | neg | neg | neg | neg | neg | neg | neg | neg |
| **L15-101** | **France/Allentown** | +++ | +++ | +++ | +++ | neg | neg | neg | + | neg | neg | neg | + | + |
| **L16-027** | **France/Allentown** | +++ | +++ | +++ | +++ | neg | neg | neg | neg | neg | neg | neg | neg | neg |
| **L16-205** | **France/Allentown** | +++ | +++ | +++ | +++ | neg | neg | neg | + | neg | neg | neg | + | + |
| **London 32** | **France/Allentown** | +++ | +++ | +++ | +++ | neg | neg | neg | neg | neg | neg | neg | neg | neg |
| **London 33** | **France/Allentown** | +++ | +++ | +++ | +++ | neg | neg | neg | + | neg | neg | neg | + | + |
| **W12-948** | **France/Allentown** | +++ | +++ | +++ | +++ | neg | neg | neg | + | neg | neg | neg | + | + |
| **W14-487** | **France/Allentown** | +++ | +++ | +++ | +++ | neg | neg | neg | + | + | neg | neg | neg | neg |
| **W15-822** | **France/Allentown** | +++ | +++ | +++ | +++ | neg | neg | neg | neg | + | neg | neg | neg | neg |
| **W15-948** | **France/Allentown** | +++ | +++ | +++ | +++ | neg | neg | neg | neg | neg | neg | neg | neg | neg |
| **EUL 4** | **France/Allentown** | +++ | +++ | +++ | +++ | neg | neg | neg | + | neg | neg | neg | + | + |
| **EUL 8** | **France/Allentown** | +++ | +++ | +++ | +++ | neg | neg | neg | + | neg | neg | neg | + | + |
| **EUL 11** | **France/Allentown** | +++ | +++ | +++ | +++ | neg | neg | neg | + | + | neg | neg | + | + |
| **EUL 12** | **France/Allentown** | +++ | +++ | +++ | +++ | neg | neg | neg | neg | + | neg | neg | + | + |
| **EUL 28** | **France/Allentown** | +++ | +++ | +++ | +++ | neg | neg | neg | neg | neg | neg | neg | + | + |
| **EUL 70** | **France/Allentown** | +++ | +++ | +++ | +++ | neg | neg | neg | neg | neg | neg | neg | + | + |
| **EUL 71** | **France/Allentown** | +++ | +++ | +++ | +++ | neg | neg | neg | neg | neg | neg | neg | + | + |
| **EUL 54** | **France/Allentown** | +++ | +++ | +++ | +++ | neg | neg | neg | neg | neg | neg | neg | + | + |
| **EUL 33-3** | **France/Allentown** | +++ | +++ | +++ | +++ | neg | neg | neg | neg | neg | neg | neg | neg | neg |
| **EUL 30** | **France/Allentown** | +++ | +++ | +++ | +++ | neg | neg | neg | + | neg | neg | neg | neg | neg |
| **W12-1057** | **Camperdown** | +++ | +++ | neg | neg | neg | neg | neg | +++ | +++ | neg | neg | neg | neg |
| **W14-826** | **Camperdown** | +++ | +++ | neg | neg | neg | neg | neg | +++ | +++ | neg | neg | + | + |
| **W14-859** | **Camperdown** | +++ | +++ | neg | neg | neg | neg | neg | +++ | +++ | neg | neg | neg | + |
| **WL 191-8** | **Camperdown** | +++ | +++ | neg | neg | neg | neg | neg | +++ | +++ | neg | neg | + | + |
| **T6-K13** | **Camperdown** | +++ | +++ | neg | neg | neg | neg | neg | +++ | +++ | + | neg | neg | neg |
| **W09-106** | **Camperdown** | +++ | +++ | neg | neg | neg | neg | neg | +++ | +++ | neg | + | + | + |
| **W09-185** | **Camperdown** | +++ | +++ | neg | neg | neg | neg | neg | +++ | +++ | neg | neg | + | + |
| **W14-834** | **Camperdown** | +++ | +++ | neg | neg | neg | neg | neg | +++ | +++ | neg | neg | + | + |
| **W14-848** | **Camperdown** | +++ | +++ | neg | neg | neg | neg | neg | +++ | +++ | neg | + | + | + |
| **T6-K13** | **Camperdown** | +++ | +++ | neg | neg | neg | neg | neg | +++ | +++ | + | neg | + | + |
| **W9-185** | **Camperdown** | +++ | +++ | neg | neg | neg | neg | neg | +++ | +++ | neg | neg | + | + |
| **W14-834** | **Camperdown** | +++ | +++ | neg | neg | neg | neg | neg | +++ | +++ | neg | neg | neg | neg |
| **WL 211-48** | **Heysham** | +++ | +++ | neg | neg | neg | neg | +++ | +++ | neg | + | + | + | + |
| **Heysham 12** | **Heysham** | +++ | +++ | neg | neg | neg | neg | +++ | +++ | neg | + | neg | neg | + |
| **Heysham 15** | **Heysham** | +++ | +++ | neg | neg | neg | neg | +++ | +++ | + | + | neg | neg | neg |
| **WL196-5** | **Heysham** | +++ | +++ | neg | neg | neg | neg | +++ | neg | neg | neg | + | neg | neg |
| **EUL 1** | **Knoxville** | +++ | +++ | +++ | +++ | +++ | neg | +++ | + | + | neg | neg | + | + |
| **EUL 19** | **Knoxville** | +++ | +++ | +++ | +++ | +++ | neg | +++ | neg | neg | neg | neg | + | + |
| **EUL 22** | **Knoxville** | +++ | +++ | +++ | +++ | +++ | neg | +++ | + | neg | neg | neg | + | + |
| **EUL 23** | **Knoxville** | +++ | +++ | +++ | +++ | +++ | neg | +++ | neg | neg | neg | neg | + | + |
| **EUL 24** | **Knoxville** | +++ | +++ | +++ | +++ | +++ | neg | +++ | neg | neg | neg | neg | + | + |
| **EUL 24** | **Knoxville** | +++ | +++ | +++ | +++ | +++ | neg | +++ | neg | neg | neg | neg | + | + |
| **EUL 29** | **Knoxville** | +++ | +++ | +++ | +++ | +++ | neg | +++ | + | neg | neg | neg | + | + |
| **EUL 66** | **Knoxville** | +++ | +++ | +++ | +++ | +++ | neg | +++ | neg | neg | neg | neg | neg | + |
| **EUL 87** | **Knoxville** | +++ | +++ | +++ | +++ | +++ | neg | +++ | neg | neg | neg | neg | + | + |
| **EUL97** | **Knoxville** | +++ | +++ | +++ | +++ | +++ | neg | +++ | neg | + | neg | neg | + | + |
| **EUL 36** | **Knoxville** | +++ | +++ | +++ | +++ | +++ | neg | +++ | neg | neg | neg | neg | + | + |
| **EUL 49** | **Knoxville** | +++ | +++ | +++ | +++ | +++ | neg | +++ | neg | neg | neg | neg | + | + |
| **EUL 63** | **Knoxville** | +++ | +++ | +++ | +++ | +++ | neg | +++ | neg | + | neg | neg | + | + |
| **WL 219-31** | **Knoxville** | +++ | +++ | +++ | +++ | +++ | neg | +++ | neg | neg | neg | neg | + | + |
| **WL 16-163** | **Knoxville** | +++ | +++ | +++ | +++ | +++ | neg | +++ | + | neg | neg | neg | + | + |
| **WL 16-170** | **Knoxville** | +++ | +++ | +++ | +++ | +++ | neg | +++ | + | + | neg | neg | + | + |
| **WL 173-1** | **Knoxville** | +++ | +++ | +++ | +++ | +++ | neg | +++ | neg | + | neg | neg | + | + |
| **WL 173-17** | **Knoxville** | +++ | +++ | +++ | +++ | +++ | neg | +++ | neg | neg | neg | neg | + | + |
| **WL 173-33** | **Knoxville** | +++ | +++ | +++ | +++ | +++ | neg | +++ | + | + | neg | neg | + | + |
| **WL 192-14** | **Knoxville** | +++ | +++ | +++ | +++ | +++ | neg | +++ | + | + | neg | neg | + | + |
| **WL 192-2** | **Knoxville** | +++ | +++ | +++ | +++ | +++ | neg | +++ | + | neg | neg | + | + | + |
| **WL 192-5** | **Knoxville** | +++ | +++ | +++ | +++ | +++ | neg | +++ | + | + | neg | neg | + | + |
| **WL 192-8** | **Knoxville** | +++ | +++ | +++ | +++ | +++ | neg | +++ | neg | neg | neg | neg | + | + |
| **WL 2013-52** | **Knoxville** | +++ | +++ | +++ | +++ | +++ | neg | +++ | + | neg | neg | neg | + | + |
| **WL 202-16** | **Knoxville** | +++ | +++ | +++ | +++ | +++ | neg | +++ | + | neg | neg | neg | + | + |
| **WL 202-17** | **Knoxville** | +++ | +++ | +++ | +++ | +++ | neg | +++ | + | + | neg | neg | + | + |
| **WL 202-7** | **Knoxville** | +++ | +++ | +++ | +++ | +++ | neg | +++ | neg | neg | neg | neg | + | + |
| **WL 202-8** | **Knoxville** | +++ | +++ | +++ | +++ | +++ | neg | +++ | neg | + | neg | neg | neg | neg |
| **WL 202-9** | **Knoxville** | +++ | +++ | +++ | +++ | +++ | neg | +++ | neg | + | neg | neg | + | + |
| **WL 204-1** | **Knoxville** | +++ | +++ | +++ | +++ | +++ | neg | +++ | + | neg | neg | + | + | + |
| **WL 204-11** | **Knoxville** | +++ | +++ | +++ | +++ | +++ | neg | +++ | + | + | neg | neg | + | + |
| **WL 204-2** | **Knoxville** | +++ | +++ | +++ | +++ | +++ | neg | +++ | neg | + | neg | neg | + | + |
| **WL 204-27** | **Knoxville** | +++ | +++ | +++ | +++ | +++ | neg | +++ | neg | + | neg | neg | + | + |
| **WL 204-30** | **Knoxville** | +++ | +++ | +++ | +++ | +++ | neg | +++ | neg | neg | neg | neg | + | + |
| **WL 204-32** | **Knoxville** | +++ | +++ | +++ | +++ | +++ | neg | +++ | neg | neg | neg | neg | + | + |
| **WL 204-49** | **Knoxville** | +++ | +++ | +++ | +++ | +++ | neg | +++ | + | neg | neg | neg | + | + |
| **WL 204-50** | **Knoxville** | +++ | +++ | +++ | +++ | +++ | neg | +++ | neg | neg | neg | neg | + | + |
| **WL 204-7** | **Knoxville** | +++ | +++ | +++ | +++ | +++ | neg | +++ | + | + | neg | neg | + | + |
| **WL 204-86** | **Knoxville** | +++ | +++ | +++ | +++ | +++ | neg | +++ | + | + | neg | neg | + | + |
| **WL 211-1** | **Knoxville** | +++ | +++ | +++ | +++ | +++ | neg | +++ | + | + | neg | neg | + | + |
| **WL 211-15** | **Knoxville** | +++ | +++ | +++ | +++ | +++ | neg | +++ | + | + | neg | neg | + | + |
| **WL 211-2** | **Knoxville** | +++ | +++ | +++ | +++ | +++ | neg | +++ | + | neg | + | neg | + | + |
| **WL 211-26** | **Knoxville** | +++ | +++ | +++ | +++ | +++ | neg | +++ | + | + | neg | neg | + | + |
| **WL 211-43** | **Knoxville** | +++ | +++ | +++ | +++ | +++ | neg | +++ | + | + | neg | neg | + | + |
| **WL 211-9** | **Knoxville** | +++ | +++ | +++ | +++ | +++ | neg | +++ | + | + | neg | neg | + | + |
| **WL 213-1** | **Knoxville** | +++ | +++ | +++ | +++ | +++ | neg | +++ | + | + | + | + | + | + |
| **WL 213-12** | **Knoxville** | +++ | +++ | +++ | +++ | +++ | neg | +++ | + | + | + | neg | + | + |
| **WL 213-15** | **Knoxville** | +++ | +++ | +++ | +++ | +++ | neg | +++ | + | neg | neg | neg | + | + |
| **WL 213-16** | **Knoxville** | +++ | +++ | +++ | +++ | +++ | neg | +++ | + | + | neg | neg | + | + |
| **WL 213-24** | **Knoxville** | +++ | +++ | +++ | +++ | +++ | neg | +++ | + | + | neg | + | + | + |
| **WL 213-30** | **Knoxville** | +++ | +++ | +++ | +++ | +++ | neg | +++ | + | + | neg | neg | + | + |
| **WL 213-36** | **Knoxville** | +++ | +++ | +++ | +++ | +++ | neg | +++ | + | + | neg | neg | + | + |
| **WL 213-37** | **Knoxville** | +++ | +++ | +++ | +++ | +++ | neg | +++ | + | + | neg | neg | + | + |
| **WL 213-50** | **Knoxville** | +++ | +++ | +++ | +++ | +++ | neg | +++ | + | + | neg | neg | + | + |
| **WL 213-54** | **Knoxville** | +++ | +++ | +++ | +++ | +++ | neg | +++ | neg | + | neg | neg | + | + |
| **WL 213-8** | **Knoxville** | +++ | +++ | +++ | +++ | +++ | neg | +++ | neg | + | neg | neg | + | + |
| **L04-412** | **Knoxville** | +++ | +++ | +++ | +++ | +++ | neg | +++ | neg | + | neg | neg | + | + |
| **L07-375** | **Knoxville** | +++ | +++ | +++ | +++ | +++ | neg | +++ | neg | + | neg | neg | + | + |
| **W03-207** | **Knoxville** | +++ | +++ | +++ | +++ | +++ | neg | +++ | neg | + | neg | neg | + | + |
| **W03-270** | **Knoxville** | +++ | +++ | +++ | +++ | +++ | neg | +++ | neg | + | neg | neg | neg | neg |
| **W03-274** | **Knoxville** | +++ | +++ | +++ | +++ | +++ | neg | +++ | neg | + | neg | neg | + | + |
| **W03-281** | **Knoxville** | +++ | +++ | +++ | +++ | +++ | neg | +++ | neg | + | neg | + | neg | neg |
| **W06-730** | **Knoxville** | +++ | +++ | +++ | +++ | +++ | neg | +++ | neg | + | neg | neg | + | + |
| **W07-120** | **Knoxville** | +++ | +++ | +++ | +++ | +++ | neg | +++ | neg | + | neg | neg | + | + |
| **W16-418-1** | **Knoxville** | +++ | +++ | +++ | +++ | +++ | neg | +++ | + | neg | neg | neg | neg | neg |
| **W16-418-2** | **Knoxville** | +++ | +++ | +++ | +++ | +++ | neg | +++ | + | + | neg | neg | neg | neg |
|  |  |  |  |  |  |  |  |  |  |  |  |  |  |  |
|  |  |  |  |  |  |  |  |  |  |  |  |  |  |  |
|  |  |  |  |  |  |  |  |  |  |  |  |  |  |  |
|  |  |  |  |  |  |  |  |  |  |  |  |  |  |  |
|  |  |  |  |  |  |  |  |  |  |  |  |  |  |  |
|  |  |  |  |  |  |  |  |  |  |  |  |  |  |  |
|  |  |  |  |  |  |  |  |  |  |  |  |  |  |  |
|  |  |  |  |  |  |  |  |  |  |  |  |  |  |  |
|  |  |  |  |  |  |  |  |  |  |  |  |  |  |  |
